# Human arm redundancy: a new approach for the inverse kinematics problem

**DOI:** 10.1098/rsos.231036

**Published:** 2024-02-28

**Authors:** Avi Barliya, Nili Krausz, Hila Naaman, Enrico Chiovetto, Martin Giese, Tamar Flash

**Affiliations:** ^1^ Motor Control for Humans and Robotic Systems Laboratory, Weizmann Institute of Science, Rehovot, Central, Israel; ^2^ Section Theoretical Sensomotorics, HIH/CIN, University Clinic of Tübingen, Tubingen, Baden-Württemberg, Germany; ^3^ Neurobotics and Bionic Limbs (eNaBLe) Laboratory, Technion—Israel Institute of Technology, Haifa, Haifa, Israel

**Keywords:** motor coordination, inverse kinematics, motion planning, motor compositionality, coordinate frames

## Abstract

The inverse kinematics (IK) problem addresses how both humans and robotic systems coordinate movement to resolve redundancy, as in the case of arm reaching where more degrees of freedom are available at the joint versus hand level. This work focuses on which coordinate frames best represent human movements, enabling the motor system to solve the IK problem in the presence of kinematic redundancies. We used a multi-dimensional sparse source separation method to derive sets of basis (or source) functions for both the task and joint spaces, with joint space represented by either absolute or anatomical joint angles. We assessed the similarities between joint and task sources in each of these joint representations, finding that the time-dependent profiles of the absolute reference frame’s sources show greater similarity to corresponding sources in the task space. This result was found to be statistically significant. Our analysis suggests that the nervous system represents multi-joint arm movements using a limited number of basis functions, allowing for simple transformations between task and joint spaces. Additionally, joint space seems to be represented in an absolute reference frame to simplify the IK transformations, given redundancies. Further studies will assess this finding’s generalizability and implications for neural control of movement.

## 1. Introduction

Human motor behaviour is extremely rich, exhibiting a large variety of possible movements. However, the question of how the nervous system plans movement remains one of the most debated issues in the field of motor control. Human arm movements are of great interest given their particular versatility and adaptability, enabling the performance of numerous different motor tasks. To a large extent this versatility is owing to the inherent kinematic redundancy of the human arm. Kinematically redundant limbs or manipulators are structures that have more degrees of freedom (d.f.) than those required for the performance of a specified task. Redundancy is highly beneficial when the need arises to overcome obstacles, adapt to environmental changes or overcome fatigue. An excess number of d.f. can allow for motor tasks to be accomplished in many different ways.

While excess d.f. can prove useful, they raise the question of how the central nervous system (CNS) overcomes the computational problems associated with the neural control of redundant d.f. Specifically, it is not fully understood how the CNS resolves kinematic redundancies to produce appropriate motor commands that can activate different limb muscles and produce forces and torques necessary to move the limb along a desired trajectory. This question was introduced by Bernstein [1] and was termed the ‘excess degrees of freedom problem’.

Redundancy, though, is not limited to the workspace or task levels. In general, the motor control hierarchy for planning movement has four levels: end-point (or task) variables (e.g. hand positions and velocities), joint angles and angular velocities, muscle activations and neural activity patterns within cortical, sub-cortical and spinal regions of the nervous system. It should be noted that redundancy appears at all of these levels. Firstly, most motor tasks can be performed using multiple possible end-effector postures and trajectories. Meanwhile, redundancy also exists at the level of joint rotations because the number of joint d.f. is larger than the number of end-effector d.f. Moreover, joint postures and rotations are controlled by generating appropriate joint torques produced by many alternative patterns of muscle activations. Owing to the overlapping muscles’ actions and the ability to co-contract more muscles than are mechanically needed, the same arm configurations can be achieved through the generation of many different muscle activation patterns, which are determined at the neuronal level. Understanding how the nervous system resolves this redundancy could also improve the control of multi-joint assistive and wearable robotic devices, such as prostheses and exoskeletons, by enabling more natural, intent-driven control that mimics the innate coordination of human movements.

Additionally, a rule of thumb is that a higher level in the motor hierarchy controls a greater number of state variables than the level below. Thus, at each level in the motor hierarchy (i.e. neural, muscular, joint and task levels) these commands or states uniquely prescribe states at the level below. For example, joint positions prescribe the hand endpoint position, and many different combinations of muscle commands can prescribe a particular joint position and limb configuration.

The inverse kinematics (IK) problem is concerned with the question of how to transform an endpoint position or trajectory specified in endpoint coordinates into joint coordinates. In the case of kinematic redundancy, the problem is how specific joint configurations can be selected (from a range of possibilities). Hence it applies to many different kinds of robots, including serial, parallel, hybrid and of course to the human arm. There have been numerous approaches described for solving this problem for serial robotic systems (given the serial design of the human arm refer to Spong *et al.* [2] for a recent review of IK solutions for serial robots). However, here we are focused on understanding how the CNS resolves kinematic redundancies to solve the human IK problem. For instance how does it define a unique arm configuration to achieve a desired hand location (in spite of the existing kinematic redundancy). Many researchers have tackled this problem and numerous approaches to finding the solution have been proposed. For example, Soechting & Terzuolo [3] addressed the IK problem in the context of elliptical hand drawing movements. Particularly, they proposed a straightforward algorithm suggesting that elliptical hand trajectories result from oscillatory patterns of joint rotations, whereby the phase shifts between the different joint oscillations were assumed to be used to determine the geometrical form and orientation of the drawing plane. Others tried to apply methods from robotics involving the use of the generalized inverse of the manipulator or arm Jacobians [4–7].

Cyclic drawing movements, however, pose some difficulty to these traditional approaches. For drawing a cyclic shape in which the end-effector returns to the initial point, these solutions produce non-repeatable trajectories in joint-space [8]. Many additional studies have tried to solve this problem of joint repeatability by suggesting a variety of IK algorithms that overcome this problem [9–15]. Meanwhile, others have focused on finding the joint-space trajectories using optimization-based methods aimed at minimizing some cost function [16–19], or using dimensionality reduction methods enabling a search for coupled or correlated d.f. [20,21].

There also has been research using optimization and computational methods as a means to explain neural motion planning. For instance, the minimum-jerk model [22] was developed to account for the kinematic characteristics of observed human hand trajectories, assuming that movement is coordinated to achieve optimal smoothness of the end-effector trajectory. Other studies have developed different models based on the minimization of alternative cost functions, such as the minimum torque-change [23] and the minimum variance [24] models.

More recently, other elaborate optimization models were developed aiming at integrating multiple costs at different levels of the motor hierarchy in order to provide a complete solution. For example, many models [25–32] have emphasized the significance of optimal feedback selection for successful generation of multi-joint movements, based on the optimal feedback control framework developed by Todorov & Jordan [33], and some others have exploited machine learning techniques [34,35].

On the other hand, a different set of approaches to the IK problem have exploited dimensionality reduction techniques. The idea behind this class of approaches is that appropriate motor commands might occupy only a subspace or manifolds of lower dimensionality within the high-dimensional space of possible solutions, involving different combinations of motor variables and control inputs [36,37]. In that case, correlations between the d.f. can be identified, and these correlations quantitatively reflect coordination patterns responsible for effectively lowering the dimensionality of the motor representation.

The law of intersegmental coordination is a good example of such an innate dimensionality reduction solution. Motor control scientists have described a behavioural phenomenon whereby the absolute elevation angles of the leg during locomotion essentially covary on a plane, known as the intersegmental plane of coordination, and can be represented by 2 d.f., instead of the three existing joint angles (hip, thigh and shank) [38,39]. The absolute elevation angles describe the orientation of the leg segments with respect to gravity as opposed to the anatomical angles that describe the orientation of one leg segment with respect to an adjacent segment. The observed covariation plane can also provide context that can be useful for understanding healthy and pathological gait patterns [40–42]. In particular, the observations described by this law of intersegmental covariation are only obtained if the joint angles are represented in terms of absolute coordinates in the sagittal plane, which is a finding that indicates the importance of identifying which reference frames subserve the representation of motor commands during human gait.

In recent years, numerous dimensionality reduction methodologies have been applied to kinematic and dynamics data obtained from studies of the motor system, resulting in different definitions of motor variables as well as units of action and motor primitives. These are usually selected based on geometrical considerations, statistical likelihoods, information theory concepts, etc. In fact, motor primitives or synergies have been shown to exist across a range of mammal and bird species [43]. However, the specific nature of these primitives are still under investigation. Specifically, in this work we have focused on the level of kinematic synergies and reformulated dimensionality reduction by using a blind source separation family of solutions. In particular, we applied a dimensionality reduction method called Fourier-based anechoic demixing algorithm (FADA) developed by the authors in [44–46] based on anechoic demixing to derive the basis functions (also called sources) that underly movement patterns. Specifically, we consider whether it may be possible to use the same single set of basis functions to represent both joint and task position variables. This would then provide a hypothesis for how the nervous system may solve the IK problem. Details of this method are presented in §2.3.

Additionally, in §2 we include a description of the arm kinematic model presented by Soechting [47], on which our study is based. Finally, in this work we focus on kinematic and mathematical analyses to determine which reference frames might allow for a unique set of basis functions to represent both joint and task spaces. Particularly, we examine the joint-space in an anatomical reference frame versus an absolute reference frame. See §2 for a longer discussion about the relevance and importance of different reference frames and representations.

## 2. Background

The goal of this work is to help solve the IK problem for arm movements using dimensionality reduction to identify basis functions that may be used by the CNS for movement coordination. An important element to consider in a given solution, is what coordinate system any solution is represented in, since this can provide insight into how the nervous system resolves redundancy. Additionally, it is important to carefully select the arm model used in obtaining results, as this may affect the solution and some arm models may allow for more impactful results. Finally, the specific method of dimensionality reduction used for identifying motor primitives or basis functions is important as some methods may produce more meaningful, generalizable results. These topics are discussed in this section.

### 2.1. Reference frames and representations

**Figure 1.**
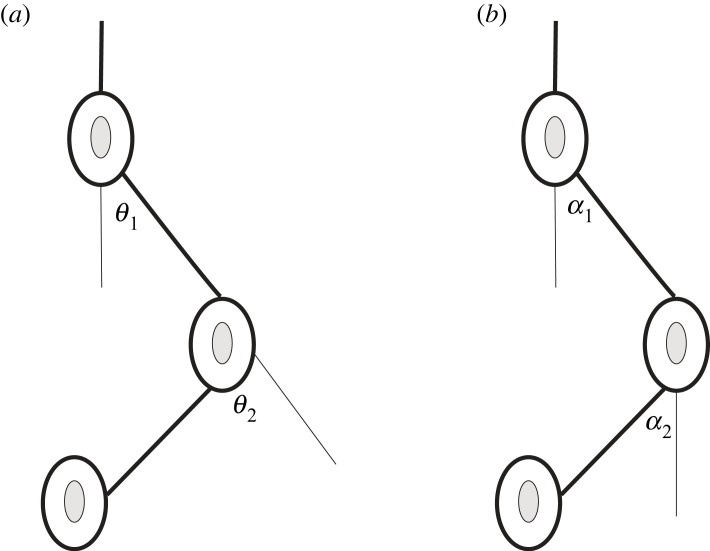
Generalized coordinates: two different representations that describe that exact same configuration: (*a*) anatomical or relative joint angles, (*b*) absolute or elevation angles.

A multi-joint system can be described by its configuration at each instant in time; however, this depends on the coordinate system used for its spatial representation. The various possible coordinate systems are termed *generalized coordinates*, which are sets of variables that uniquely and compactly define the configuration of the system (coordinates in the configuration space). Figure 1 exemplifies two possible coordinate systems for a simple kinematic chain. Theoretically, one could define an infinite number of coordinate system *representations*, but it turns out that these representations can be grouped into specific classes. Two representations in the same class are equivalent if a rigid/linear transformation between them exists. Therefore, two questions should be asked for a given system: (i) can the controlled representation be identified? and (ii) what are the reasons for choosing one representation over another?

The question of representation is critical in motor control. Many studies have focused on which variables (kinematic, dynamic, etc.) are represented and controlled by the CNS for movement planning and execution. Over the years, this question has attracted considerable attention and among other studies, Soechting and Flanders and colleagues have extensively studied this issue. Specifically, in Soechting & Ross [48] they conducted several psychophysical studies aimed at examining alternative representations to determine which particular representation subserves the control of human arm posture and movement. Their experiments consisted of a *matching task* in which one arm was set to a given joint angle by the experimenter and the subject was asked to match this joint angle with their other arm. Movement in the matching arm was constrained to the d.f. being investigated. Their working hypothesis was that the ‘natural’ coordinate representation of joint angles would have the lowest standard deviation in the difference between the joint angles of the two limbs.

**Figure 2.**
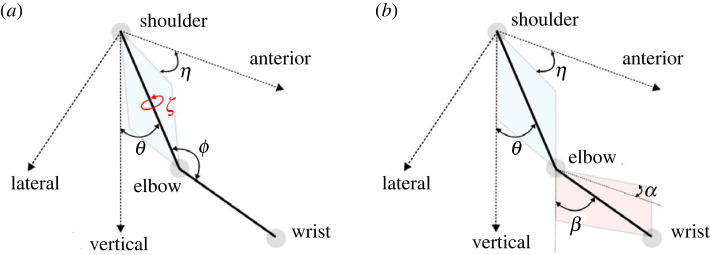
Arm model. In (*a*) the first representation (*θ*, *η*, *ζ*, *ϕ*) combining absolute and internal reference frames is shown and in (*b*) the second representation (*θ*, *η*, *β*, *α*) with a completely external reference frame is shown. The plane of the upper arm is shown in blue and the plane of the forearm is shown in pink.

In Soechting [47], the authors investigated three different coordinate systems for the shoulder orientation and two different systems for the elbow, and according to their psychophysical results discovered that the sense of limb orientation appears to be expressed best by the coordinate system illustrated in figure 2*b*, picking (*θ*, *η*, *α*, *β*) to represent the arm configuration. It should be noted that rather than using relative orientation, such as defining the forearm joint angle with respect to the upper arm (*ϕ*), these angles describe the limb orientation relative to an absolute frame of reference.

Borghese *et al.* [39] investigated coordination patterns of leg segments and joints during locomotion. Importantly, they examined leg joint coordination in two sets: anatomical (relative) joint angles and elevation angles (absolute). In their paper, they claim that these two representations form different classes and no simple transformation exists between them. The hip angle can be used to illustrate this point. The thigh elevation angle is the angle between the thigh and the vertical, and the hip angle is the angle between the thigh and the torso. Thus the hip angle cannot be reconstructed unless the torso is assumed to be vertical. Therefore, the transformation between coordinate frames without torso orientation is not trivial.

Furthermore, it was shown in multiple studies [38,39,49] that elevation angles are well behaved, have highly sinusoidal properties and when plotted with respect to each other, they lie on a plane during the gait cycle. On the other hand, the time course of the anatomical angles is much more variable, including inter-cycle variability. Additionally, Soechting & Ross [48] also found that subjects were best able to match joint angles of their right and left arms when they were measured relative to the vertical axes and the sagittal plane.

In particular, these experiments identified yaw and elevation angles as the selected spatial coordinate system for the perception of arm orientation. Studies have shown that target location is initially defined in a reference frame centred at the eyes and the origin of this reference frame is shifted toward the shoulder during the neural processing for targeted arm movements [50]. Then, in this shoulder-centred frame of reference, target location is defined by three parameters: distance, elevation and azimuth [51]. Moreover, it was suggested by Flanders & Soechting [52] that there exists a linear transformation involving two separate *channels*: arm elevation is computed from target distance and elevation, and arm yaw is computed from target azimuth.

In another study, Ivanenko *et al.* [53] suggested that there might be independent control of parameters in spherical representation of the end effector. In particular, they used principal component analysis (PCA) applied to elevation angle data and found a strong correlation between the PCA-derived basis functions of absolute angle basis functions and those of the polar and radial coordinates.

These studies have all contributed to our understanding of how the nervous system represents and coordinates movements. For instance, it seems that the nervous system has some preference for an absolute frame of reference, and yaw and elevation angles seem to be keys to joint coordination. The validation of these insights is essential. To help validate these coordinate frame insights, in this work FADA will be used to find sources (basis functions) in both joint-space, represented by both the relative/anatomical and the absolute/orientation angles, and in task-space, represented by the end-point Cartesian coordinates. Additionally, there still remain open questions related to the coordination of movement. Importantly, the intersegmental coordination of the arm has been neglected for the most part in the literature, which is a primary focus of this study.

### 2.2. Arm model

To model the arm, we first can consider that an unconstrained rigid object in three-dimensional space has 6 d.f. in total: three translational and three rotational. However, when there are multiple linked segments the number of d.f. is reduced owing to kinematic constraints. For example, assuming no translational movements at the shoulder (glenohumeral joint), the upper arm has 3 d.f., which can be modelled by a ball-and-socket joint allowing flexion/extension, abduction/adduction and internal/external rotations. The forearm, modelled as a hinge joint, adds two more d.f.: flexion/extension, and pronation/supination. Thus, there are a total of 5 d.f. for the simplified human upper limb (more if we consider the wrist and fingers). However, in the current study, we used a simpler model for the arm (i.e. not including elbow pronation/supination), with two rigid links joined at the elbow joint, ending with only 4 d.f.

Specifically, our arm model and the angles we used as defined in Soechting *et al.* [54] is illustrated in figure 2. In this model, the first angular rotation (*η*) is about the *z*-axis and determines the yaw angle. The second angular rotation (*θ*) is about an axis perpendicular to the arm plane (this is the plane spanned by the vectors of the upper-arm and forearm, the arm plane is the lateral *x*-axis if there is zero yaw) and determines the arm’s elevation. The third angular rotation (*ζ*) is about the humeral axis. This rotation does not change the elbow location but does affect the wrist location in space and the arm plane. Finally, *ϕ* is the angle of flexion of the forearm, *ϕ* = 180° corresponding to full extension.

It should be noted that figure 2*b* contains two more angles: *β*, the angular elevation of the forearm, and *α*, the forearm yaw angle (just like *η* for the upper-arm). Thus, two sets of variables, or representations, can be devised to fully describe the arm configuration. The first representation (*θ*, *η*, *ζ*, *ϕ*) combines external (absolute) and internal (relative) reference frames (figure 2*a*). In this case, *θ* represents the upper-arm elevation angle, *η* is the azimuth angle, *ζ* the humeral rotation angle, and the elbow flexion–extension angle is given by *ϕ*. The second representation (*θ*, *η*, *β*, *α*) is given in a completely external frame of reference (figure 2*b*). Here, *β* and *α*, denote the elevation and azimuth of the upper-arm, respectively. We term the first representation *anatomical* or *relative*, and the second is termed *absolute* or *external*.

### 2.3. Dimensionality reduction

Movement data (such as joint angles or muscle activations) generally have high dimensionality. However, regardless of the level of complexity, every arm movement is ultimately mapped to three Euclidean coordinates describing the hand position. Therefore, a set of correlations or a coordination pattern must exist in these higher motor levels that constrains any seemingly excess d.f. The challenge is to identify these correlations.

One of the most well-known methods for identifying coordination patterns is the PCA method. This method involves a mathematical procedure that maps possibly correlated variables onto a small set of *uncorrelated* variables called principal components. The basic underlying assumption for PCA is that the observed data (*x*_*i*_(*t*)) can be modelled as a linear combination of orthonormal basis functions (*s*_*j*_), the vectors of which are the eigenvector of the covariance matrix representing the data, with time-dependent mixing coefficients for the principal components (PCs).

Another well-known method for dimensionality reduction is independent component analysis (ICA). In contrast to PCA, the underlying model assumed by ICA uses a time-dependent basis functions *s*_*j*_(*t*) with constant mixing coefficients. More importantly, here the basis is required to be independent, rather than uncorrelated as for PCA, which is a stricter constraint. ICA has classically been used as a solution to the *cocktail-party problem* [55]. This problem is a special case of the *blind source separation* (BSS) problem.

An alternate version of the BSS problem is anechoic blind source separation. Similarly to ICA, this method aims to solve the BSS problem, while allowing for the addition of time delays between the sources:

2.1 $$x_{i}(t)=\sum_{\;j=1}^{n}\alpha_{ij}s_{j}(t-\tau_{ij})\;i=1,\ldots ,m.$$

The model expressed in equation (2.1) appears in acoustic equations where a reverberation-free environment is modelled, i.e. the sensors only receive attenuated sounds with different arrival times. Thus, mixtures of the form (2.1) have been termed *anechoic mixtures*. A solution for a system assuming a nonlinear model such as described in equation (2.1) was proposed by Omlor & Giese [56]. The solution assumes that the sources are uncorrelated and uses properties of the stochastic Wigner–Ville spectrum [57]. The solution was obtained by representing the signals in the time-frequency domain using the Wigner–Ville transform, which is defined by

2.2 $$Wf\;(x,\omega )\;:=\int E\left\{\;f\left(x+\frac{t}{2}\right)\bar{\;f\left(x-\frac{t}{2}\right)}\right\}\;e^{-2\pi i\omega t}\;dt,$$

where E denotes the expected value and the bar denotes the complex conjugate. Applying this transformation to equation (2.1) and exploiting the independence of the sources (this condition is only approximately fulfilled by the computational algorithms used), one obtains

2.3 $$Wx_{i}(\eta ,\omega )=|\alpha {|}_{ij}^{2}Ws_{j}(\eta -\tau_{ij},\omega ).$$

Assuming that the observed data coincide with the mean of the distribution (*x*_*j*_ ≈ *E*(*x*_*j*_)) one can compute the zero moment of equation (2.3) and obtain the following two equations:

2.4 $$\left|Fx_{i}{|}^{2}(\omega )=\sum_{j}^{n}|\alpha {|}_{ij}^{2}|Fs_{j}{|}^{2}(\omega \right)$$

and

2.5 $$|Fx_{i}(\omega ){|}^{2}\cdot \frac{\partial }{\partial \omega }\arg\{Fx_{i}\}=\sum_{j}^{n}|\alpha {|}_{ij}^{2}\cdot |Fs_{i}{|}^{2}\cdot \left[\frac{\partial }{\partial \omega }\arg\{Fs_{j}\}+\tau_{ij}\right]$$

where F is the Fourier transform operator. Non-negative ICA was used to solve equation (2.4), the results of which are used in equation (2.5) in order to extract the corresponding time delays. The latter was solved in an iterative manner as detailed in the above mentioned papers. Therefore, the solution of the above system of equations results in the requested set of sources (*s*_*j*_) and the corresponding time delays (*τ*_*ij*_).

In this study in both the FADA and anechoic mixture decomposition algorithms the sources are real by construction. In the FADA algorithm, for example, it is ensured that the reconstructed sources are real by guaranteeing that the Fourier coefficients correspond to positive and negative frequency pairs that are always complex conjugates of each other (see Chiovetto *et al.* [46], eqn (8) and in the following discussion.)

This method models high-dimensional signals as a linear superposition of a small set of source functions, which can have additional fixed temporal delays. The solution results in a set of sources for each space to which it was applied. Then, for each movement type the proper mixing weights and time delays were determined. Thus, this approach can allow for a simple computational solution to the redundancy problem, based on the assumption that the same basis functions underly the joint and task spaces.

Here, a new approach to resolving the redundancy problem was suggested that exploits dimensionality reduction called FADA [45], which is based on a computationally efficient form of anechoic demixing algorithm for the analysis of band limited signals (Omlor & Giese [44]). This was originally derived from a more general anechoic demixing algorithm developed by Omlor & Giese [56]. FADA is a highly efficient dimensionality reduction approach and an alternative way to characterize motor primitives based on the idea that they express invariance across time. In particular, in this work we use FADA to produce basis functions (or sources) for either the task or joint spaces.

This can help researchers gain a greater understanding of how the CNS possibly generates desired task space trajectories with appropriate movements of redundant joint d.f. Specifically, FADA provides basic temporal patterns or functions *s*_*p*_(*t*) which are combined or superposed to reconstruct a set of temporal signals. Hence, the temporal decomposition is mathematically described as:

2.6 $$x_{m}^{l}(t)=\sum_{\;p=1}^{n}=c_{mp}^{l}\cdot s_{p}(t-\tau_{mp}^{l})+\text{residuals.}$$

In this equation, $x_{m}^{l}(t)$ is the value of the *m*th d.f. at time *t* in trial number *l*, and the corresponding scalar mixing weights $c_{mp}^{l}$ change between trials of different types (experimental conditions), and *p* signifies the total number of temporal primitives. This model also allows for time shifts between the temporal basis functions for different d.f., which are captured by the variables $\tau {\;}_{mp}^{l}$. The time delays and linear mixing weights are typically assumed to vary over trials, while it is assumed that the basis functions *s*_*p*_(*t*) are invariant across trials [45]. Details of the iterative FADA algorithm are described in more detail in §3.3.

## 3. Material and methods

### 3.1. Motion capture and data processing

Arm movements were recorded for 15 subjects (aged 28–35) who volunteered for the experiment. None reported previous hand injuries and all gave their informed consent prior to their inclusion in the study.

Subjects were grouped based on the Chiovetto motion sets they completed: one group had four subjects who each conducted all motion types (ALL), one group had six subjects who only completed the figure eight motion (FE) and one group had five subjects who only completed the planar ellipse motion (PE), though at different orientations in space. For future reference, these groups will be referred to as ALL, FE and PE, respectively.

**Figure 3.**
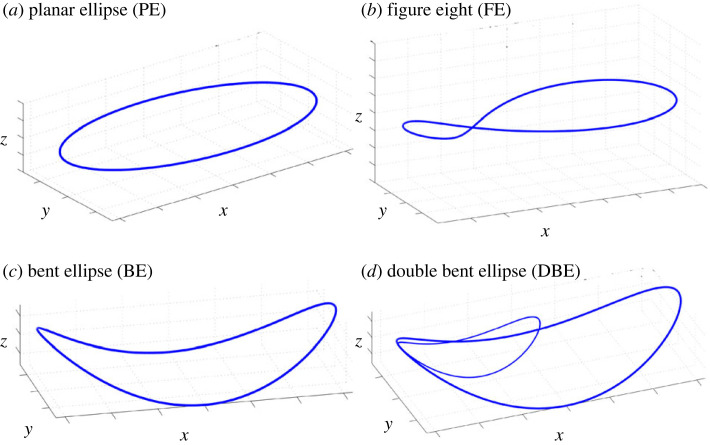
Wire frame models of the recorded shapes, as shown to subjects prior to data collection. These were the main shapes, but the FE and PE shapes were also collected at different orientations.

Subjects were instructed to freely draw these series of shapes in three-dimensional space repetitively at a comfortable pace with their dominant arm. Ten paths were drawn by the subjects in the ALL group: planar ellipse (PE), vertical ellipse drawn on the sagittal plane (PE-V), ellipse drawn on the frontal plane (PE-F), planar ellipse drawn on a plane rotated $45^{\circ }$ off the sagittal plane (PE-45V), ellipse drawn on a plane rotated $45^{\circ }$ off the frontal plane (PE-45F), horizontal figure-eight (FE), vertical figure-eight drawn on the sagittal plane (FE-V), figure-eight drawn on the frontal plane (FE-F), bent-ellipse (BE), double bent-ellipse (DBE) and up-down movements (UD). The models of the shapes are presented in figure 3. The drawing instructions did not involve the experimenter demonstrating the movements to avoid biasing the subjects towards a specific behaviour. Therefore, we prepared wire frames models for the different shapes and showed them to the subjects before the recordings of each shape began.

**Figure 4.**
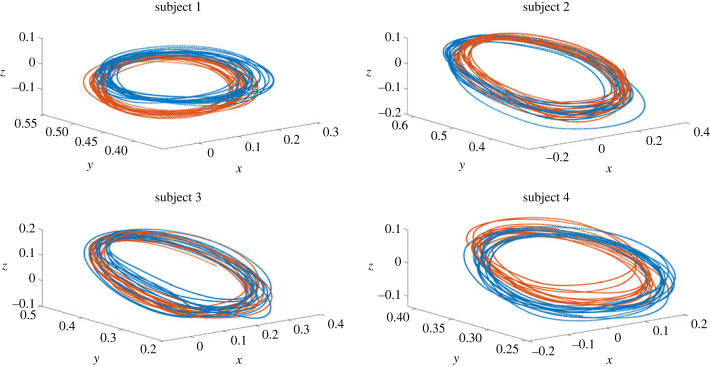
Data of the planar ellipse (PE) shape for the four subjects from the ALL group. Each subject completed two 20 s trials, shown in (1) blue and (2) orange. Note variability in the traces for each subject.

Subjects were seated on a wooden chair with a high rigid back rest. Movements of the ALL group were recorded using a Polhemus ‘Liberty’ electromagnetic spatial tracking system where sensor positions and orientations were collected at 240 Hz and preliminary experiments were conducted to measure the accuracy of the tracking system, and the error was found to be at most 0.3 mm. Eight sensors were positioned on the arm: one on the wrist, two on the forearm so as not to be co-linear with the wrist marker, two on the upper-arm, one on the shoulder (again, ensuring these three sensors are not co-linear), one on the chest near the collarbone, and one on the chair acting as reference. The tracing of each shape was recorded continuously for 20 s. For the ALL group two 20 s trials were carried out for each shape per subject. Thus, the database of movement for the ALL group consists of 4 (subjects) × 10 (shapes) × 2 (trials per subject) = 80 recordings (see example PE data in figure 4).

Meanwhile for the PE and FE groups, movements were recorded using a Vicon IR motion capture system. We analysed the data using a simplified arm model in which the end-effector is at the centre of the wrist, the task space has 3 d.f. and the configuration space has 4 d.f. (three in the shoulder and one in the elbow). In each trial, subjects had to draw the shape for 25 s with their healthy, dominant right hands.

The data were interpolated for missing samples, then approximated by smooth analytic curves (to allow high-order differentiation). The continuous data were then segmented into individual repetitions. Though the use of two different motion tracking systems could produce slight differences between the subject groups, we believe it should not significantly impact the results of our source reference frame or timing comparisons.

### 3.2. Joint angle extraction

Joint angle calculation is not a trivial task. First, one has to identify the centers of rotation of the arm located at the shoulder and elbow, and in some cases it is not possible or simple to place a marker exactly on the joint centre. This problem is crucial to the study of human movement and biomechanics, including the calculation of joint angles. Many approaches have been used in the literature to try to address this problem. We have chosen to use an extension of the technique that was described in Gamage & Lasenby [58].

### 3.3. Source separation

While there are many methods of performing source separation such as PCA and ICA, in this work we used the FADA algorithm as first presented in Chiovetto *et al.* [45]. This algorithm can allow for source separation without constraints but is also flexible to allow for parameter non-negativity or other constraints.

Specifically, this iterative algorithm is as follows:

(i) non-negative ICA is used to solve the equation:
  3.1 $$|c_{rk}{|}^{2}=\sum_{u=1}^{U}|a_{ru}{|}^{2}|v_{uk}^{2}|,$$
  with *r* = 0, 1, …, *R* and *k* = 0, 1, …, *K*. Non-negative matrix factorization [34,41] can also be used to solve this equation instead of ICA.
  Specifically here, the mixing weights are represented by *a*_*ru*_, while *c*_*rk*_ and *v*_*uk*_ represent the coefficients of the Fourier expansion of the *r*-th joint angle and *u*th temporal source signals, respectively;
(ii) to obtain the phase shifts, the Fourier coefficients are then updated by solving the nonlinear least-square equation:
  3.2 $$\min_{\Phi }\|C-Z(\Phi ){\|}_{F}^{2},$$
  where *F* is the Frobenius norm and **C** and **Z** are given as **C**_*rk*_ = *c*_*rk*_ and
  $$Z_{rk}=\sum_{u=1}^{U}a_{ru}\;e^{ik\tau_{uk}}|v_{uk}|\;e^{i\phi v_{uk}};$$
(iii) the weights *a*_*ru*_ and delays *τ*_*ru*_ are then identified for each signal *y*_*r*_(*t*), by minimizing the cost function:
  3.3 $$\underset{a_{r},\;\tau_{r}}{\mathrm{argmin}}\|y_{r}(t)-f(t,\;\tau_{r}{)}^{\prime }a_{r}{\|}_{F}^{2}.$$

Here the source functions *f*_*u*_(*t*) are kept constant (and assumed to be uncorrelated), and it is assumed the time delays are independent. Specifically, the vector **f**(*t*, *τ*_*r*_) is the concatenation of these functions including the time shift associated with each d.f. *r*.

### 3.4. Inverse kinematics

In this section, we show that given two arbitrary spaces (possibly of different dimensionalities) that share a similar set of basis functions, one can transform between the spaces using the basis functions as mediators. This idea was originally proposed based on some of our previously obtained results. Specifically, we would like to show that there is a mechanism to determine the joint-space trajectory given a desired trajectory in task-space, and assuming that the task-space and joint-space movements share the same set of sources. We begin by formally defining the problem itself.

#### 3.4.1. Problem statement

As mentioned, we assume a nonlinear model underlying both the joint and task-spaces. Thus, the basis functions can also be shifted in time. Let the *shift operator* *U*_*λ*_, applied on a periodic function *f*, be defined as follows:

3.4 $$(U_{\lambda }f)(t)\;:=f\;(t-\lambda ).$$

The shift operator *U* essentially delays the function *f*(*t*) by *λ* in a circular periodic manner. This is actually achieved through the *convolution* operation:

$$(U_{\lambda }f)(t)=\int_{R}\delta (t-\lambda -s)f\;(s)\;ds,$$

where *δ*(*x*) is the Dirac delta function. The above definition is for the one-dimensional case, and next we define a more complex operator for the multidimensional case.

Let $\bar{f}={\left[f_{1}(t),\;f_{2}(t),\;f_{3}(t)\right]}^{T}$ be a vector of periodic functions (the extracted sources). We define the *shift operator matrix* as follows:

3.5 $$T=\left(\begin{array}{ccc} U_{\lambda_{11}} & U_{\lambda_{12}} & U_{\lambda_{13}}\\ U_{\lambda_{21}} & U_{\lambda_{22}} & U_{\lambda_{23}}\\ U_{\lambda_{31}} & U_{\lambda_{32}} & U_{\lambda_{33}} \end{array}\right),$$

where $U_{\lambda_{ij}}$ are the standard shift operators as defined above (equation (3.4)). Then, applying T on $\bar{f}$ we get,

$$(T\bar{f})(t)=\left(\begin{array}{c} \left(U_{\lambda_{11}}f_{1})(t)+(U_{\lambda_{12}}f_{2})(t)+(U_{\lambda_{13}}f_{3})(t\right)\\ \left(U_{\lambda_{21}}f_{1})(t)+(U_{\lambda_{22}}f_{2})(t)+(U_{\lambda_{23}}f_{3})(t\right)\\ \left(U_{\lambda_{31}}f_{1})(t)+(U_{\lambda_{32}}f_{2})(t)+(U_{\lambda_{33}}f_{3})(t\right) \end{array}\right)$$

⇓

$$(T\bar{f})(t)=\left(\begin{array}{c} f_{1}(t-\lambda_{11})(t)+f_{2}(t-\lambda_{12})(t)+f_{3}(t-\lambda_{13})(t)\\ f_{1}(t-\lambda_{21})(t)+f_{2}(t-\lambda_{22})(t)+f_{3}(t-\lambda_{23})(t)\\ f_{1}(t-\lambda_{31})(t)+f_{2}(t-\lambda_{32})(t)+f_{3}(t-\lambda_{33})(t) \end{array}\right).$$

Thus, the *problem* is to define the inverse of T ($T^{-1}$) such that if we define $\bar{b}$ as:

$$\bar{b}(t)=(T\bar{f})(t),$$

then applying $T^{-1}$ will result in

$$(T^{-1}\bar{b})(t)=\bar{f}(t).$$

The problem can be slightly extended to the *weighted* version of the operator T. Let,

3.6 $$T_{w}=\left(\begin{array}{ccc} a_{11}U_{\lambda_{11}} & a_{12}U_{\lambda_{12}} & a_{13}U_{\lambda_{13}}\\ a_{21}U_{\lambda_{21}} & a_{22}U_{\lambda_{22}} & a_{23}U_{\lambda_{23}}\\ a_{31}U_{\lambda_{31}} & a_{32}U_{\lambda_{32}} & a_{33}U_{\lambda_{33}} \end{array}\right),$$

where *a*_*ij*_ are weights. Applying $T_{w}$ on $\bar{f}$ is just

$$(T_{w}\bar{f})(t)=\left(\begin{array}{c} a_{11}f_{1}(t-\lambda_{11})(t)+a_{12}f_{2}(t-\lambda_{12})(t)+a_{13}f_{3}(t-\lambda_{13})(t)\\ a_{21}f_{1}(t-\lambda_{21})(t)+a_{22}f_{2}(t-\lambda_{22})(t)+a_{23}f_{3}(t-\lambda_{23})(t)\\ a_{31}f_{1}(t-\lambda_{31})(t)+a_{32}f_{2}(t-\lambda_{32})(t)+a_{33}f_{3}(t-\lambda_{33})(t) \end{array}\right).$$

So similarly to the previous inverse, $T_{w}^{-1}$ should be defined. In effect, having such an inverse applied on, say the task-space trajectory, we will be able to obtain the sources in return.

#### 3.4.2. Simplified case (without time delays)

We begin with a simpler version of the above problem, in which the sources are not shifted in time, i.e. *λ*_*ij*_ = 0. Let *s* = (*s*_1_, *s*_2_, *s*_3_) be the vector of the extracted sources. The sources are assumed to be identical for both spaces, thus the joint angles, *q*, can be expressed as

3.7 $$q_{4xp}=A_{4x3}{\left(\begin{array}{c} s_{1}\\ s_{2}\\ s_{3} \end{array}\right)}_{3xp}=As^{T}.$$

Similarly, using the same sources the Euclidean coordinates, *x*, can be expressed as

3.8 $$x_{3xp}=B_{3x3}{\left(\begin{array}{c} s_{1}\\ s_{2}\\ s_{3} \end{array}\right)}_{3xp}=Bs^{T},$$

where *A* and *B* are the matrices of amplitudes associated with the sources, and *p* is the number of points in the path.

In order to attend to the first case of a direct mapping between joint and task-spaces through the forward kinematics (*f*_*i*_ : **Q** → **X**), observe that the dimension of *B* is 3 × 3. Thus, in a well-behaved case and relying on the independence of the sources, *B* can be regarded to be invertible and therefore

3.9 $$B^{-1}x=s^{T}.$$

Substituting *s* from equation (3.9) into equation (3.7) we obtain:

3.10 $$q=AB^{-1}x.$$

This is an inverse mapping of positional variables (not velocities). Note that although the sources *s* were the original mediators, they do not appear in the transformation and only their amplitudes are required.

The second case, involving the mapping of instantaneous positions is a bit more complicated. Specifically, the mapping between joint and end-effector velocities (or instantaneous position) for forward kinematics is given by:

3.11 dr=J dq ,

with *J*(*q*) as the Jacobian of the forward kinematics.

Using equations (3.11) and (3.7) can be re-written as follows:

3.12 $$\dot{x}=J(q)\frac{d}{dt}q=J(q)\frac{d}{dt}(As^{T})$$

and

3.13 $$\dot{x}=J(q)\left[\left(\frac{d}{dt}A\right)s^{T}+A\frac{d}{dt}s^{T}\right],$$

since

$$\frac{d}{dt}A=0$$

and

3.14 $$\dot{x}=F{\dot{s}}^{T},$$

where *F* = *J*(*q*)*A* is of dimensions 3 × 3 and potentially invertible.

This takes the form of a coordinate transformation into a space that can be uniquely inverted to the task-space. Thus the first derivative of the joint angles, $\dot{q}$, can be expressed as a linear combination of a new set of sources $\dot{s}$. We find this has the form

3.15 $$\dot{q}=K{\dot{s}}^{T},$$

where *K* is a matrix containing the coefficients of ${\dot{s}}^{T}$, and *K* = *AB*^−1^*F*.

So far though, the solution is only for the degenerate case where time delays are ignored. This is not a realistic state, and it will be remedied in the next section.

#### 3.4.3. Inverse solution

Next, we address how to integrate the required time shifts into the inverse transform, using a circular shift operator.

A convolution operator *A* is formally defined as

3.16 $$(Af)(t)=\int_{R}k(t-s)f\;(s)\;ds,\;t\in R.$$

The function *k* in this context is referred to as the *convolution kernel* of *A*. Let $F\;:\;L^{2}(R)\to L^{2}(R)$ denote the *Fourier transform*:

3.17 $$(Ff)(x)\;:=\hat{\;f}(x)\;:=\int_{R}f\;(t)\;e^{itx}\;dt,\;x\in R$$

and let $F^{-1}\;:\;L^{2}(R)\to L^{2}(R)$ be the inverse of F, given by

3.18 $$(F^{-1}g)(t)=\frac{1}{2\pi }\int_{R}g(x)\;e^{-itx}\;dx,\;t\in R.$$

The operator (3.16) can formally be written in the form

3.19 $$A=F^{-1}\hat{k}F,$$

or equivalently, $(Af)\;\hat{\;}(x)=\hat{k}(x)\hat{\;f}(x),x\in R$. The function $\hat{k}$ is called the *symbol* of the operator (3.16) and (3.19).

Now we consider a special class of convolution operators on *L*^2^(**R**).

Fix *λ* ∈ **R** and let $U_{\lambda }$, a bounded linear operator,^1^ be the *shift operator* defined by

$$(U_{\lambda }f)(t)\;:=f\;(t-\lambda ),\;t\in R.$$

since

$$(U_{\lambda }f)\;\hat{\;}(x)=\int_{R}f\;(t-\lambda )\;e^{itx}\;dt=\int_{R}f\;(s)\;e^{i(s+\lambda )x}\;ds=\;e^{i\lambda x}\hat{\;f}(x).$$

Using the Dirac delta function *δ*(*t*), we have

3.20 $$(U_{\lambda }f)(t)=\int_{R}\delta (t-\lambda -s)f\;(s)\;ds,\;t\in R,$$

which shows that $U_{\lambda }$ is a convolution by the kernel *δ*(*t* − *λ*). The convolution operator is often expressed as a binary operation between a function *f* and a kernel *k* denoted by

$$f\;*\;k=\int_{R}f\;(s)k(s-t)\;ds.$$

Therefore, according to the above definitions and properties, it is clear that the convolution commutes with the shift operator (translation), that is

$$U_{\lambda }(f\;*\;g)=(U_{\lambda }f)\;*\;g=f\;*\;(U_{\lambda }g).$$

To connect this to our problem, we assume that each signal can be represented a follows:

$$z_{i}(t)=\sum_{\;j=1}^{3}a_{ij}s_{j}(t-\tau_{ij}).$$

Let us assume that a similar set of sources underlie the joint-space and task-space and representing the joints and hand behaviour by equations (3.7) and (3.8), respectively. Consider first the representation of a one-dimensional signal in terms of the convolution and shift operators:

$$z(t)=\sum_{i=1}^{3}a_{i}s_{i}(t-\tau_{i})=\sum_{i=1}^{3}a_{i}s_{i}(t)\;*\;\delta (t-\tau_{i})$$

⇓

$$z(t)=\sum_{i=1}^{3}a_{i}(U_{\tau_{i}}s_{i})(t),$$

where $U_{\tau_{i}}$ is the shift operator described in equation (3.20). We can now extend the one-dimensional case to a multidimensional space.

Let

3.21 $$x_{i}(t)=\sum_{\;j=1}^{3}a_{ij}s_{j}(t-\tau_{ij}),\;i=1,\ldots ,3,$$

where *x*_*i*_ are the Euclidean coordinates of the hand. Thus, the hand coordinates can be represented by a mixture (sum) of the weighted (*a*_*ij*_) and delayed (by *τ*_*ij*_) sources (*s*_*j*_(*t*)). Equation (3.21) can be expressed differently using the convolution operator, as follows:

3.22 $$x_{i}(t)=\sum_{\;j=1}^{3}a_{ij}s_{j}(t)\;*\;\delta (t-\tau_{ij}),$$

where *δ*(*t*) is the Dirac delta function, and the symbol * stands for convolution. Now, applying the Fourier transform on both sides of equation (3.22), we obtain

3.23 $$F[x_{i}(t)](\xi )=F\left[\sum_{\;j=1}^{3}a_{ij}s_{j}(t)\;*\;\delta (t-\tau_{ij})\right](\xi ),$$

where F is the fourier transform defined by equation (3.17). Owing to the linearity of the Fourier transform, equation (3.23) can be rewritten as

$$F[x_{i}(t)](\xi )=\sum_{\;j=1}^{3}a_{ij}F[s_{j}(t)\;*\;\delta (t-\tau_{ij})](\xi )$$

⇓convolution theorem

3.24 $$F[x_{i}(t)](\xi )=\sum_{\;j=1}^{3}a_{ij}F[s_{j}(t)](\xi )\cdot F[\delta (t-\tau_{ij})](\xi ).$$

The Fourier transform of the delta function is

3.25 $$F[\delta (t-\tau_{ij}](\xi )=\int_{-\infty }^{\infty }\delta (t-\tau_{ij})\;e^{-2\pi it\xi }\;dt=\;e^{-2\pi i\tau_{ij}\xi }.$$

Substituting equation (3.25) into equation (3.24) we get:

3.26 $$F[x_{i}(t)](\xi )=\sum_{\;j=1}^{3}a_{ij}F[s_{j}(t)](\xi )\cdot \;e^{-2\pi i\tau_{ij}\xi }.$$

The system of equations (3.26) is the frequency domain version of the temporal domain system (3.21). In this domain, the inverse is more easily expressed.

Let the matrix

3.27 $$A_{ij}(\xi )=a_{ij}\;e^{-2\pi i\tau_{ij}\xi },$$

be the matrix that contains the information of the mixing coefficients of the sources and the their corresponding time-delays. Then, system (3.26) can be expressed in matrix form as

3.28 $$\left[\begin{array}{c} F[x_{1}(t)](\xi )\\ F[x_{2}(t)](\xi )\\ F[x_{3}(t)](\xi ) \end{array}\right]=A(\xi )\left[\begin{array}{c} F[s_{1}(t)](\xi )\\ F[s_{2}(t)](\xi )\\ F[s_{3}(t)](\xi ) \end{array}\right],$$

where *A*(*ξ*) = [*A*_*ij*_(*ξ*)] is a 3 × 3 matrix. Then, by inverting *A* we get,

$$\left[\begin{array}{c} F[s_{1}(t)](\xi )\\ F[s_{2}(t)](\xi )\\ F[s_{3}(t)](\xi ) \end{array}\right]=\underset{b={\left(b_{1},\;b_{2},\;b_{3}\right)}^{T}}{\underbrace{A^{-1}(\xi )\left[\begin{array}{c} F[x_{1}(t)](\xi )\\ F[x_{2}(t)](\xi )\\ F[x_{3}(t)](\xi ) \end{array}\right]}}=\left[\begin{array}{c} b_{1}(\xi )\\ b_{2}(\xi )\\ b_{3}(\xi ) \end{array}\right].$$

Applying the inverse Fourier transform on both sides of the above system leads to

$$\left[\begin{array}{c} F^{-1}[F[s_{1}(t)](\xi )](t)\\ F^{-1}[F[s_{2}(t)](\xi )](t)\\ F^{-1}[F[s_{3}(t)](\xi )](t) \end{array}\right]=\left[\begin{array}{c} F^{-1}[b_{1}(\xi )](t)\\ F^{-1}[b_{2}(\xi )](t)\\ F^{-1}[b_{3}(\xi )](t) \end{array}\right]$$

⇓

3.29 $$\left[\begin{array}{c} s_{1}(t)\\ s_{2}(t)\\ s_{3}(t) \end{array}\right]=\left[\begin{array}{c} F^{-1}[b_{1}(\xi )](t)\\ F^{-1}[b_{2}(\xi )](t)\\ F^{-1}[b_{3}(\xi )](t) \end{array}\right],$$

and we have the solution for the inverse problem. The solution here, is having the sources themselves extracted as a function of the task-space.

An important note to mention is that the solution above assumes that the matrix *A*(*ξ*) is invertible. The matrix *A* might not be always invertible, but its properties can be analysed and this has been considered and presented in the electronic supplementary material, appendix A. This analysis ultimately showed that *A* is invertible if and only if |*A*| ≠ 0.

Thus far, we have shown that provided two spaces share a basis it is possible to go back and forth between the spaces. In fact, the proof itself illustrates this methodology. Furthermore, the CNS could hypothetically use this to solve the IK problem using the basis functions as mediators between the spaces. However, there are some knowledge gaps left to fill. Assume that we are planning a movement in task-space, and would like to produce the corresponding joint-space trajectory. The results of the previous section state that the joint-space trajectory can be expressed by:

3.30 $$q_{i}(t)=\sum_{\;j=1}^{3}\beta_{ij}s_{j}(t-{\tilde{\tau }}_{ij}),$$

where *β*_*ij*_ and ${\tilde{\tau }}_{ij}$ are the appropriate weights and time shifts, respectively. Furthermore, similarly to the process in the previous section which concluded in equation (3.27), the time delays ${\tilde{\tau }}_{ij}$ can be pulled out of the sources and be represented. Then, *s*_*j*_ can be replaced with equation (3.29), thus *q*_*i*_(*t*) is expressed in terms of *x*_*i*_(*t*) (the task-space):

$$\begin{array}{c} s(t)\equiv s(t,x)\\ ⇓\\ q_{i}(t,x)=\sum_{\;j=1}^{3}\beta_{ij}s_{j}(t-{\tilde{\tau }}_{ij},x). \end{array}$$

Indeed, the above solution shows how the joint-space Q(t) trajectories can be expressed in terms of the task-space trajectories X(t). However, the parameters *β*_*ij*_ and ${\tilde{\tau }}_{ij}$ are not an outcome of the above process and need to be determined. In the next section, we will investigate the sources, the source-based reconstruction, and the source phase shifts, which will provide us with clues on how the time delays are determined.

### 3.5. Source analysis

Each shape is described by a set of task space sources and a set of joint space sources. These sources can be compared in three major ways: (i) by analysing how similar the source shapes are to one another, (ii) by assessing the effectiveness of the reconstruction of the end effector trajectory produced by the sources, and (iii) by analysing how the sources are shifted in time, or phase, between each other.

#### 3.5.1. Source shape analysis

The joint-space sources (both representations) were compared with those of the task-space both visually and numerically (see figure 8 for description). To aid visual comparison between the sources they were normalized using their $L_{2}$ norms. Then, the cross-correlation was computed between the different sources (each source in joint-space was cross-correlated with all other sources in the task-space) to both find a match between sources and shift them in time with respect to each other to overlay the sources in one plot. The cross-correlation between two curves *f* and *g* is defined as

$$(f⋆g)(\tau )\overset{\text{Def}}{=}\int_{-\infty }^{\infty }f^{*}(\tau )g(t+\tau )\;d\tau ,$$

where f* denotes the complex conjugate of *f*.

The cross-correlation similarity measure index (similarity index or SI for short), defined as the maximum correlation value between two signals, was compared between each task-space source and its joint-space counterpart, given by the anatomical and absolute orientation-angle sources. For very similar signals, values that are close to 1 will be achieved at zero (or close to zero) time lag.

We performed a two-way ANOVA test to determine if the differences between the task and joint space sources were statistically significant, while accounting for the differences because of to subjects and selected movement shapes. The factors that were considered for our ANOVA were the angle type (absolute versus anatomical angle), the shape (i.e. FE versus PE, etc.) and the subject. Subject was considered a random factor and was nested within the shape group, as some subjects only completed movements of a single shape. The null hypothesis in this case was that there was no difference in the source correlations (i.e. neither set of joint space sources was more closely correlated to the task space sources). The results of this analysis would simply signify that there is a significant difference in the source correlations, but the magnitude and direction of that difference would need to be analysed more carefully using other methods and post-hoc comparisons.

We also used the sign test to evaluate if there are statistically significant differences between sources. This is analogous to a non-parametric *t*-test, with no assumptions of a normal distribution. In particular, the sign test looks at whether one signal (or in this case, one source) is statistically larger or smaller than another. This is achieved by forming a vector where each entry indicates if the corresponding entry for one signal, i.e. source one, is smaller than the matching entry for signal two, in this case source two. The null hypothesis here is that the distributions are equal, and therefore there is an equal likelihood for entries from one signal to be larger than entries in the other signal. The proportion of entries in the comparison vector that indicate one source being smaller than the other can be compared using binomial distribution tables. For two matching sources, the proportion would be 0.5.

So we use a normal approximation of the binomial distribution to calculate the *z*-score. The *z*-score of the data could then be compared to the *z*-score of the null probability (we made sure that the estimated probability *p* and the size of the data *n* were consistent with *n* · *p* > 5 and *n* · (1 − *p*) > 5.) to determine the *p*-value. Values of *p* < 0.05 were considered significant and indicated that the sources were not statistically equivalent.

#### 3.5.2. Source-based trajectory reconstruction

Since our methods use the sources to allow for transforming between task and joint spaces, this means we can use them to reconstruct the end-effector trajectory (or task-space trajectory), and by comparing the original data to the reconstructed data we can quantify the quality of our source-based reconstruction. In particular, we computed a reconstruction accuracy, based on the following equation:

3.31 $$\text{accuracy}=1-\frac{\mathrm{norm}{\left(x_{\mathrm{rec}}-x_{0}\right)}^{2}}{\mathrm{norm}{\left(x_{0}-\bar{x}\right)}^{2}},$$

where *x*_rec_ is the reconstructed trajectory, *x*_0_ is the original trajectory, $\bar{x}$ is the mean of the original trajectory, and the norm is given as the frobenius norm. Here the closer of a value to 1, the better the reconstruction accuracy.

#### 3.5.3. Source phase-shift analysis

Each movement cycle for an individual shape is distinct. This distinction becomes apparent when considering the weights and the time-delays of the sources that generate the movement. We performed an analysis to represent patterns of the time-delays (*τ*_*ij*_), assuming that these are not arbitrarily determined, and to expose correlations between the time-delays for different sources, i.e. phase-shifts. Note that the terms time-delay and phase-delay are sometimes used interchangeably depending on whether we are considering the time or frequency domain, where a time-delay is multiplied by 2*π* to compute the phase-delay. In particular, we will carry out the time-delay analysis for the absolute joint-space and task-space representations. Phase-shifts for three-dimensional shapes and planar shapes will be separately considered.

Consider the following system:3.32
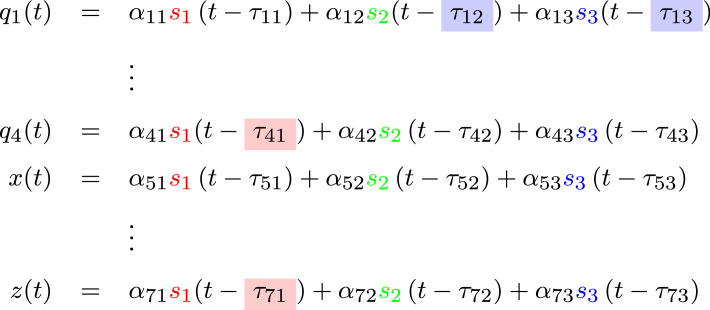
The phase-shifts can be analysed across two levels. Firstly, phase-shifts between sources of the same d.f. were examined, i.e. *τ*_*ij*_ − *τ*_*ik*_ (see for example the blue blocks in equation (3.32): *τ*_12_ − *τ*_13_). Secondly, phase-shifts between different d.f. in a single source were examined, i.e. *τ*_*ij*_ − *τ*_*kj*_ (for example, red blocks in equation (3.32): *τ*_41_ − *τ*_71_). Constant phase-shifts of the former type, if appearing, may imply coordination between different sources. One can think of the sources as movement generators, and thus constant phase-shifts would be the result of coordination at the level of these generators.

The second type of phase-shifts can appear owing to coordination between different d.f., such as joint level coordination. The phase-shifts are computed as the absolute value difference between the sources’ phases, corrected to their relative percentage of one drawing cycle (|φ_*i*_ − φ_*j*_|/*p*, where *p* is the number of sample points in a movement cycle). Specifically, since we normalize the data to be 200 points in length for each cycle, the phase-shifts values can range between 0 and 200 in normalized (unitless) time. In the results, we will present the relative percentage of drawing cycle by dividing the delays by 200, to produce a percentage deviation from the entire cycle.

## 4. Results

In this section, we will show the results of the source separation for our data.

### 4.1. Source shape analysis results

Sources were extracted from recorded data from all subjects. Visual inspection of these sources can provide insight into the significance of the anatomical and absolute angles.

#### 4.1.1. Individual source examples

**Figure 5.**
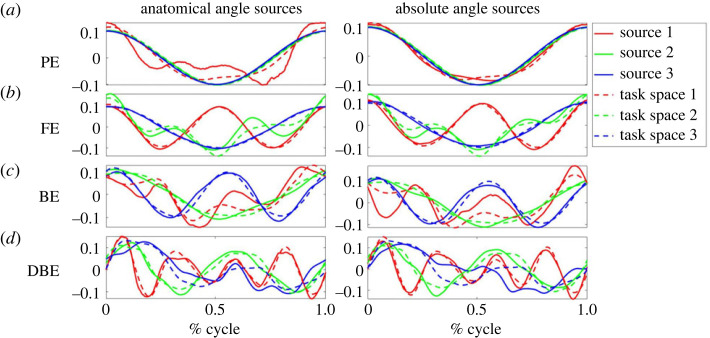
Sample source results from a typical subject from the ALL test group. Example sources are shown for the four shape types: (*a*) PE or planar Ellipse, (*b*) FE or figure eight, (*c*) BE or bent ellipse, (*d*) DBE or double bent ellipse. For each shape, sources based on the anatomical angles (shown on the left) and sources based on absolute angles (shown on the right) are compared to the end effector task space sources (see dashed lines). Using visual inspection alone, it is clear that the absolute angle sources generally provide a better fit for the task space sources in this example, particularly for the PE and FE cases.

**Figure 6.**
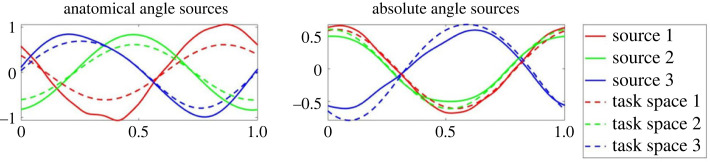
Sample source results for a typical subject from the PE test group. As shown in figure 5 the anatomical angle sources are shown on the left and the absolute angle sources are shown on the right, both compared to the end effector task space sources (shown in dashed lines). Note that the shape is similar to that of the PE sources for the ALL subject. However, the sources are phase shifted compared to the consistent cosine graph shape of the ALL subject.

**Figure 7.**
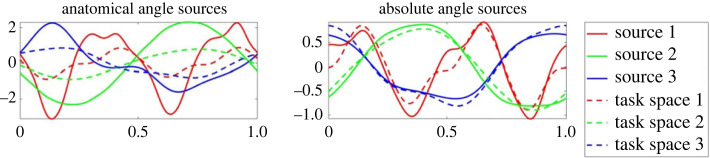
Sample source results for an FE test group subject. Note differences between the shapes of these sources and those for the FE shape of the ALL example subject.

In figure 5 example source results for each shape are shown for a typical subject in the ALL test group (for group definitions see §3.1 on the motion capture). Meanwhile, figures 6 and 7 present example source results for individual subjects from the PE and FE groups, respectively. Across all subject groups the absolute angle sources appear more similar to the end effector sources than the anatomical angle sources.

Note that the source shapes are somewhat affected by the orientation of the arm movements during the experiment. For instance, the PE group were not constrained to draw ellipses on planes of precise orientation (and thus there was variability in the orientation of movements combined to find the sources), whereas the sources for the PE shapes from the ALL group were collected at precise orientations for which separate sources were computed. We believe this is the reason that there is a phase shift between sources of the PE group and the sources for the PE shape from the ALL group, even though the shape of the sources are similar (figures 6 and 7).

#### 4.1.2. Overall source shape results

**Figure 8.**
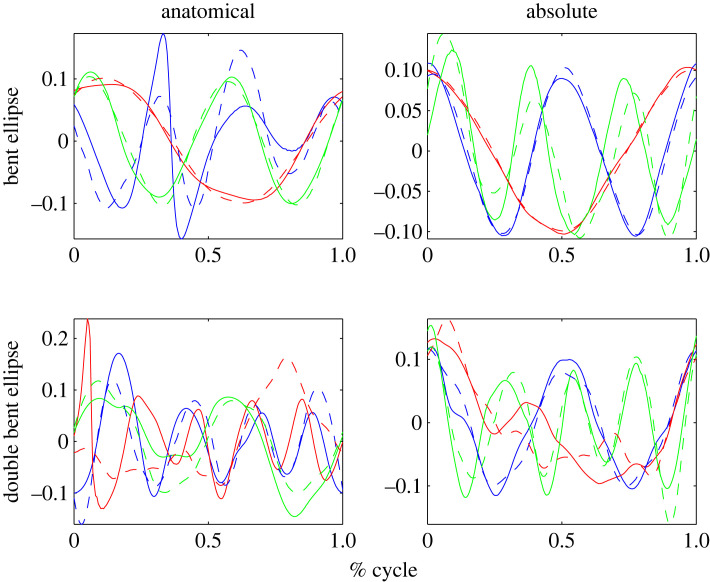
Extracted sources for the bent ellipse (top) and double bent ellipse (bottom) shapes: the left column shows results for the joint-space using an anatomical representation, the right column shows results for the joint-space using an absolute representation. The task-space sources are overlaid over the sources of the anatomical angles representation (left column) and over the absolute angles representation (right column). Solid lines represent joint-space sources and dashed lines designate task-space sources.

Figures 8 through to 10 show the sources from each recorded shape, as extracted from the recorded data for all subjects. For each shape, three sources were extracted for both joint representations as well as for the task-space and overlays of the task-space extracted sources are shown on top of the sources extracted from the joint-space (with the left column showing the anatomical sources and the right showing the absolute).

**Figure 9.**
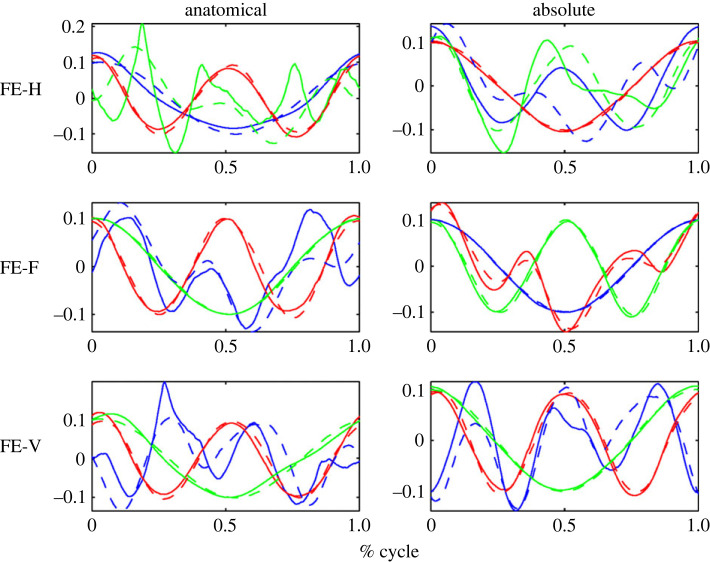
Extracted sources for the figure eight shapes at different orientation. FE-H is horizontal, FE-F is on the frontal plane, FE-V is on the sagittal plane. The rest of the details are as in figure 8.

**Figure 10.**
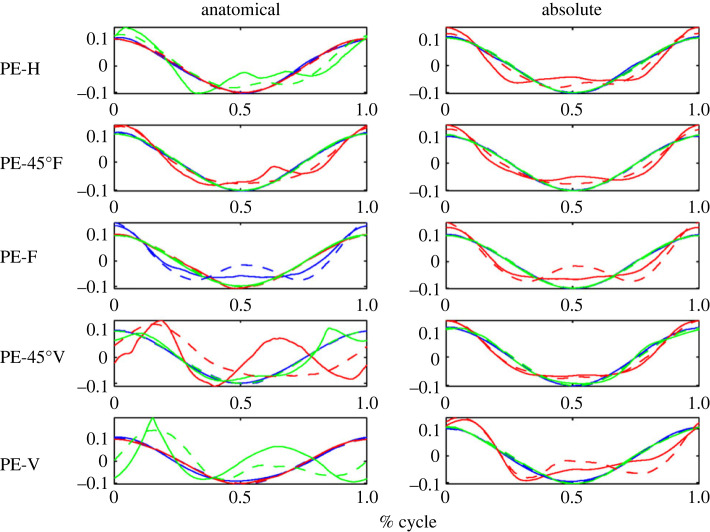
Extracted sources for the planar ellipses shapes at different orientations. PE-H is a horizontal ellipse, PE-45°F is tilted at 45° with respect to frontal plane, PE-F is on the frontal plane, PE-45°V—45° with respect to sagittal, and PE-V is on the sagittal plane. The rest of the details are as in figure 8.

Inspecting the overlays of the sources reveals a striking similarity between the task-space sources and the joint-space sources when represented by the absolute angles (orientation). There is a high degree of matching between these two sets of sources, and potentially one set of sources could be used to explain both the task-space and the joint-space when represented in its absolute orientation form.

This observation is supported by the cross-correlation similarity index values between the sources as they appear in table 1. See §3.5.1 for explanation of how the similarity index is computed. This table shows the difference between the similarity index for the absolute angle sources compared to its counterpart end-effector (task-space) sources and the similarity index for the anatomical angle and end-effector sources. In particular, a distinction was made between the cases when the absolute angle sources had higher similarity to the end-effector sources than the anatomical angle sources (as signified by the positive values, shown in dark green). The results show that in most cases the absolute angle sources were more highly correlated with the task-space sources than were the anatomical angle sources, as signified by the values greater than zero (with higher values signifying greater differences in similarity indices between the two source types).

**Table 1.** Correlation differences. (The cross correlation was computed between the anatomical angle sources and the task space sources, and a second cross correlation was computed between the absolute angle sources and task space sources. The difference between these two cross correlation measures are presented here. The last column reports the differences between the means of the correlation indices. Positive values (shown in dark green) represent cases where the absolute angles have higher correlation with the task sources. Negative values (shown in light green) represent instances where the anatomical angles have higher correlation with the task sources.)

	*S*_1_	*S*_2_	*S*_3_	mean
FE X0 Y0	0.1010	0.026	–0.16	–0.011
FE X90 Y0	0	0.2240	0.0070	0.770
FE X0 Y90	0.007	0.011	0.085	0.034
PE X0 Y0	0.001	0.054	0	0.018
PE X45 Y0	0	0.009	0	0.004
PE X90 Y0	0	0.001	–0.005	–0.002
PE X0 Y45	0.615	0	0.034	0.217
PE X0 Y90	0.36	0	0.008	0.122
BE	0.005	0.1460	0.006	0.052
DBE	0.099	0.71	0.114	0.308

As discussed in §3, the sign test and ANOVA statistical analyses were also conducted to ascertain statistically whether the anatomical or absolute sources were more similar to the end-effector coordinate sources.

The sign test used a normal approximation of the binomial distribution to calculate the *z*-score that would result for a probability of 0.5. This is equivalent to there being no difference between the two distributions. The results of the sign test yielded a *p*-value of *p* = 0.0018, which was statistically significant, assuming *α* = 0.05. Therefore, we conclude that there is a statistically significant difference between pairs of observations, which in this case represent the anatomical and absolute angle sources.

Meanwhile, the results of our two-way ANOVA are shown in table 2. The *p*-values for ‘type’ (i.e. absolute versus anatomical angles) and for ‘shape’ (i.e. PE, FE, DBE or BE) were 0.0054 and 2.95 * 10^−7^, respectively, therefore these two factors are significant. Meanwhile, the random factor ‘subject’, which is nested within the shape factor, had a *p*-value of 0.2674, meaning this factor was not significant.

**Table 2.** Results of ANOVA. (The *p*-values for the fixed reference frame and shape factors are shown, as well as for the subject factor which is nested within shape.)

	type	shape	subject (shape)
*p*-values	0.0054	2.95 * 10^−7^	0.2674
factor	fixed	fixed	random

#### 4.1.3. Source-based reconstruction accuracy

**Figure 11.**
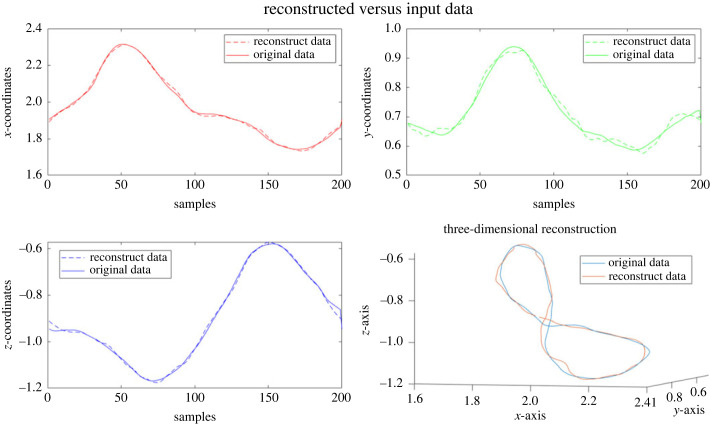
Example of data reconstruction. Here we see an example trial that has been reconstructed using the sources, with comparison to the original data trial. As is clear, though not perfect, the sources do a good job of reconstructing the movement trajectory.

The results of the end effector trajectory reconstruction were computed for the different shapes and subject groups. In figure 11, an example trial is presented. Here the original *x-*, *y-* and *z*-values of the end effector trajectory are shown, along with the source-based reconstruction. Additionally, a three-dimensional reconstruction is shown for this figure eight drawing.

An analysis of the accuracy of the reconstructions is shown in table 3. Here the accuracy of the reconstructions are given for each shape, as a number from 0 to 1, with higher values meaning greater accuracy. The mean and standard deviations across all subjects and trials are shown, with the best results being given by the planar shapes, PE and FE, and the worst accuracy produced for the DBE shape. Note that the accuracies were found to be essentially unchanged when using the relative or absolute angle sources, so here the average is shown.

**Table 3.** Reconstruction accuracy. (The accuracy of the reconstruction of the end effector trajectory, based on the sources generated by FADA. Specifically, we compare the accuracy between the reconstructed trajectory to the original data trajectory. The higher the mean value, the better the sources are able to reconstruct the original data results. Here we see that the best results are given for the purely planar movements, particularly in the FE and PE only groups, and that the worst reconstruction is given by the DBE group. However, in all we see very high accuracy of reconstruction across shapes and groups.)

shape	mean	s.d.
PE only	0.9975	7.73 × 10^−4^
FE only	0.9940	0.0044
BE	0.931	6.28 × 10^−4^
DBE	0.7826	0.0053
FE X0 Y0	0.9599	0.0011
FE X0 Y90	0.9088	0.0012
FE X90 Y0	0.9465	7.63 × 10^−4^
PE X0 Y0	0.9777	7.09 × 10^−4^
PE X0 Y45	0.9804	9.01 × 10^−4^
PE X0 Y90	0.9639	8.63 × 10^−4^
PE X45 Y0	0.9759	0.0012
PE X90 Y0	0.9811	8.44 × 10^−4^

### 4.2. Source phase-shift results

Here we present results of the time-delay analysis for the absolute joint-space representation and the task-space, and show that the time delays (*τ*_*ij*_) are not arbitrarily determined and expose correlations between the time delays, i.e. phase-shifts. Noteworthy results are highlighted. Essentially, the drawn shapes can be partitioned into three-dimensional shapes (bent-ellipse or double bent ellipse) and planar shapes. An example figure showing the BE phase shifts is shown here, but see the electronic supplementary material, appendix B for the results of the source phase-shifts for other shapes. The average results are summarized in table 4.

**Table 4.** Average phase-shifts and s.d. (±) for the planar horizontal ellipse (PE-H), ellipse drawn in the sagittal plane (PE-V), ellipse drawn in the frontal plane (PE-F), ellipse drawn on a $45^{\circ }$ slanted plane with respect to the sagittal (PE-$45^{\circ }$V) and ellipses drawn on a $45^{\circ }$ slanted plane with respect to the frontal plane (PE-$45^{\circ }$F). (The values in the parentheses stand for the s.d. as percentage of one cycle duration.)

	horizontal			vertical		
shift	*η* − *α*	*η* − *x*	*α* − *x*	*θ* − *β*	*θ* − *z*	*β* − *z*
BE	72.1 ± 4.2	85.4 ± 2.9	13.3 ± 3.04	50.7 ± 5.1		50.1 ± 1.9
	(2.1%)	(1.4%)	(1.5%)	(2.6%)		(1.0%)
DBE	80.0 ± 10.2	85.1 ± 8.5	7.0 ± 4.8	n.a.		
	(5.1%)	(4.3%)	(2.4%)			
FE-H	95.3 ± 3.6	95.4 ± 3.6	4.0 ± 2.3	n.a.		
	(1.8%)	(1.8%)	(1.2%)			
FE-V	n.a.			90.7 ± 7.7	4.4 ± 2.8	94.7 ± 6.7
				(3.8%)	(1.4%)	(3.3%)
FE-F	91.7 ± 4.8	93.7 ± 3.9	3.1 ± 1.6	50.3 ± 3.3		50.2 ± 1.5
	(2.4%)	(2.0%)	(0.8%)	(1.7%)		(0.8%)
PE-H	69.3 ± 23.5	75.6 ± 16.2	15.7 ± 8.1	n.a.		
	(11.8%)	(8.1%)	(4.1%)			
PE-V	n.a.			76.0 ± 23	15.6 ± 14	78.4 ± 21.6
				(11.5%)	(7.0%)	(10.8%)
PE-F	17.4 ± 15.6	50.3 ± 15.6	9.4 ± 13.0	74.5 ± 18.0	11.4 ± 14.7	27.2 ± 11.5
	(7.8%)	(7.8%)	(6.5%)	(9.0%)	(7.3%)	(5.8%)
PE-$45^{\circ }$V	49.9 ± 18.7	64.3 ± 32.9	13.7 ± 8.5	87.7 ± 12.8	10.7 ± 9.8	92.5 ± 5.4
	(9.4%)	(16.5%)	(4.2%)	(6.4%)	(4.9%)	(2.7%)
PE-$45^{\circ }$F	49.5 ± 16.8	19.7 ± 13.8	27.2 ± 17.5		12.4 ± 14.1	48.1 ± 12.6
	(8.4%)	(6.9%)	(8.8%)		(7.1%)	(6.3%)

#### 4.2.1. Three-dimensional shapes

**Figure 12.**
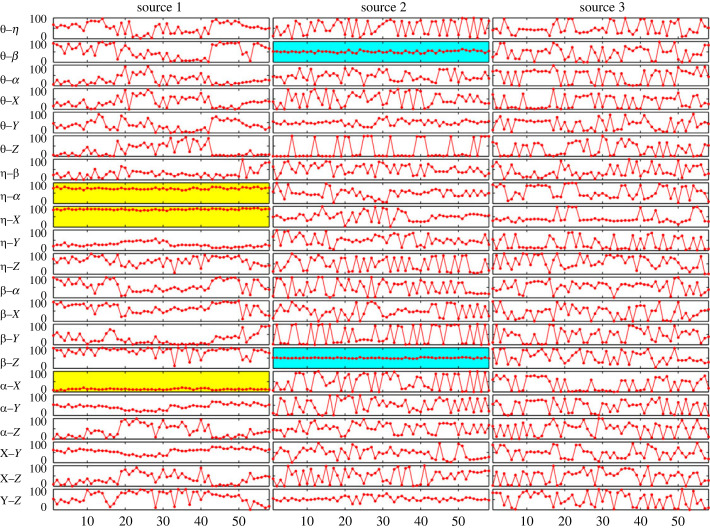
Bent ellipse (BE) phase-shifts. The plot shows the phase shifts between the azimuth angle of the upper arm, *η*, azimuth angle of the forearm, *α*, elevation angle of the upper arm, *θ*, and elevation angle of the forearm *β*. Each source is represented by one column. For each source, all possible phase-shifts between the d.f. are presented in rows. Each point in the plot represents one movement cycle. The horizontal axes denote the number of recorded cycles. Yellow and blue highlight behaviour of interest, namely similar phase-shifts for all subjects. Yellow designates lateral-related variables and blue highlights vertical-related variables.

We begin by observing the phase-shifts for the bent-ellipse shape (figure 12). Interestingly, all subjects demonstrate similar behaviour when inspecting certain phase-shifts. A constant phase-shift (72.08 ± 4.23 s.d.^2^ ) between the azimuth angles of the upper-arm and the forearm (*η* − *α*) appears in the first source (*s*_1_).

Similarly, the phase-shifts between the upper-arm’s azimuth angle and the lateral direction (*η* − *x*) and the forearm’s azimuth angle and the lateral direction (*α* − *x*), are constant for all subjects (highlighted with yellow in figure 12; electronic supplementary material, Appendix B). Moreover, the second source (*s*_2_) exhibits phase locking between the upper-arm and forearm elevation angles (*θ* − *β*) and between the elevation angle of the forearm and the upwards direction (*β* − *z*) for all subjects (highlighted in blue in figure 12).

The values of these phase-shifts are summarized in table 4. It should be noted that these values can range between 0 and 200, since one normalized cycle for each shape contains 200 sample points. Thus, although the standard deviations in the table appear in units of sample points, it is better to view them as percentage of one cycle duration in order to assess how large they are. This means that standard deviations of 10 signify 5% of a cycle duration. That is, the standard deviations for the BE shape appearing in table 4 are quite low and generally in the range of approximately 3% and less.

Therefore, two main observations should be made. First, similar phase-shifts for all subjects are maintained not only within task or joint spaces, e.g. *η* − *α* but also across the spaces, e.g. *η* − *x*. Second, one source (*s*_1_) maintains the phase-shifts among the azimuthal-related variables, whereas another source (*s*_2_) does the same for the elevational-related variables. It is as if the variables of movement are distributed between two channels of control. One for azimuth and one for elevation. The third source does not seem to play a role in the azimuth or elevation control, but it may reflect the variations between the cycles and subjects.

This phenomenon repeats itself for the other shapes, however not always to its full effect as in the case of the bent-ellipse drawing. When we observe the results for the double bent ellipse drawing (electronic supplementary material, figure B.1), we see that the azimuth-related variables are phase locked, just like for the bent-ellipse shape. Again, the constant phase-shifts for the lateral (azimuth) related variables are handled by only one source (*s*_2_). However, the same is not obtained for the vertical related variables (also see table 4).

#### 4.2.2. Planar shapes

When inspecting planar shapes, one can further divide these into shapes that contain movement only in one principal plane, (i.e. horizontal or sagittal) and shapes that are drawn on slanted planes which include movements in both the horizontal and vertical directions. The differences in the drawing planes observed in the type of phase-locks that emerge will be shown next. When drawing the figure eight shape in the horizontal plane (for context see the electronic supplementary material, appendix B: figure B.3) one source (*s*_3_) maintains a phase lock among the azimuthal related variables.

This can be explained simply by the fact that there is no vertical component to the horizontal figure eight shape. Table 4 summarizes the phase shifts for the horizontal figure 8 in row FE-H.

Similarly, as would be expected, when drawing the figure eight in the sagittal plane, only vertical related variables are coupled (electronic supplementary material, figure B.4). These phase-shifts can be seen in table 4 in row FE-V.

However, when the figure eight is drawn in the frontal plane, so that the shape has both lateral and vertical components, an identical phase locking pattern to that of the bent ellipse shape is observed (compare figure 12 and electronic supplementary material, figure C.3). The corresponding phase-shifts can be viewed in table 4 in row FE-F. Once again, the s.d. in table 4 should be considered as percentage of one period of a cycle. Thus, the s.d. for these phase-shifts are mostly approximately less than 3% of a cycle period.

Next, results for the planar ellipse are shown. In addition to the principal planes (horizontal, frontal and sagittal), ellipses were also drawn on $45^{\circ }$ slanted planes off the sagittal and frontal planes. The results for an example planar ellipse shape can be considered in the electronic supplementary material, appendix B: figure B.2. Although similar behaviour as before can be detected, the variance values of the phase-shifts are higher with respect to the other shapes as can be observed in table 4. That is, behaviour during ellipse drawing was less consistent than for other shapes, with standard deviations of roughly 7.5% of one cycle period. It is not clear yet why results for the planar ellipse are worse than for the other cases. One possibility stems from the fact that the ellipse shape is not a constraining shape. This fact may have led to larger variations in the arm configurations while the subjects were drawing this shape.

## 5. Discussion

The question of how the nervous system controls and coordinates multi-joint movements is essential within neuroscience, as well as extremely important for the development of a variety of robotic applications including humanoid robots and including rehabilitation robots and techniques that can ultimately improve function and movement abilities for individual subjects suffering from a variety of impairments. In this work, we were mainly concerned with the kinematic redundancy of the human arm. Specifically, we wanted to better understand how the nervous system controls the motions of different upper-limb segments in joint-space to achieve a desired arm trajectory executed in task-space. This is far from a trivial question, since the excess d.f. available in the joint space compared to the three-dimensional task-space d.f. renders the solution-space to be of very high dimensionality.

Thus, this work has two main results. First, we have shown that using a specific decomposition scheme we can identify sources (functional basis functions) that are highly similar for both task and joint spaces. This finding particularly holds when the joint space is represented in terms of an external reference frame (absolute angle representation). Second, we have shown that movement composition from such basic sources and using the same temporal primitives at the task and joint level could serve as a mechanism to help solve the IK problem in the presence of kinematic redundancies. Furthermore, these results have shown that the set of sources, although shifted in time with respect to each other, are not arbitrarily shifted but are prescribed by the couplings (or coordination) between different d.f. These results and future directions are discussed below.

### 5.1. Dimensionality reduction

The sources for the joint and task-spaces were strikingly similar based on the results obtained from using the FADA source separation method. However, this similarity between the two source sets was very strong when the joint space was represented in an external frame of reference (or absolute angles/coordinates), but significantly less strong when the joint-space was represented in anatomical/relative coordinates. It should be noted that this dimensionality reduction scheme is *unsupervised* and no assumptions were made with respect to the functional form of the sources.

The fact that we were able to represent the joint-space with a similar set and number of sources as those of the task-space (for the movement analysed here this number was found to be three for both coordinate frames) means not only that the intrinsic dimensionality of the joint-space is the same, but that these spaces share some geometrical properties and structure. Since these two different spaces share a single set of sources, we were able to show that one can go back and forth between the joint and task spaces. The sources themselves can serve as ‘mediators’ (channels) between the configuration and task spaces. This phenomenon might be used by the CNS as a mechanism to solve the IK problem.

It should be noted that the suggested solution is general, in the sense that it does not require the underlying sources to have specific properties. The only requirement is that the joint and task spaces share the same set of sources. As long as this requirement is satisfied, they can be integrated into the IK solution regardless of what those sources look like or what method is used to compute the sources. Thus, the solution is independent of the specific methodology used for acquiring them. However, further mathematical analysis and modelling may enable us to more rigorously define the nature of these basis functions. Furthermore, the required time-shifts for both sets of sources need to be identified. We know that these time-shifts (or phase shifts) are not arbitrary and are constrained by the specific shapes that are drawn and by the duration of the movements in task space. It is worth mentioning that an earlier study by Barliya *et al.* [49] has shown that it is possible to account for the intersegmental constraint during human locomotion by assuming that each segment of the human leg follows a simple oscillatory pattern with certain phase shifts with respect to the other segments. Here a different mathematical approach was used by the FADA scheme also involves using Fourier series but yielding more economical mode of representation in the form of combined modes.

Importantly, to assess the performance of the source-separation method we reconstructed the end effector trajectories from the original data and compared the reconstructed data to the original data using a reconstruction accuracy measure. In the case of planar movements, we found very high accuracy reconstruction, with an average of over 99% accuracy for subjects who only performed the planar motions, and upwards of 95% for all other planar groups, even those that were non-horizontal with the exception of the FE X0 Y90 case. Though in the case of the non-planar movements the accuracy was somewhat degraded (with the lowest mean accuracy being the DBE), this is not surprising, and may point to the need for a greater number of sources for the reconstruction of three-dimensional movements. However, we maintain our current approach since for the majority of our movement data three sources seemed to be sufficient. Further study of this point may be considered. Overall, though the results were very promising, showing a high degree of reconstruction using our FADA source method.

### 5.2. Phase-shifts and coordination

The specific decomposition scheme used here allowed us to derive the time delays among the different sources used to describe the same d.f. as well as the phase shifts between the onset times of the same source for different d.f. This allowed us to examine phase synchronicity among different d.f. (e.g. joints, or task-level direction and a joint rotation) based on the difference between their time delays and phase shifts between different sources corresponding to the same d.f. Importantly, using this approach the individual time delays of each source in joint-space need to be determined to produce the correct trajectories in that space. We have shown that different d.f. both in the joint-space and across the joint and task spaces are *coordinated* with specific time-delays. For a specific pairing of variables, we showed that specific phase-shifts (time delays) exist for all subjects and trials.

Specifically, we have shown that variables related to lateral movement (azimuth) tend to be represented by one source, and variables related to the elevation (i.e. vertical) are coordinated by another source. Indeed, this result comes from a completely computational process. However, this may reveal elements of the way the motor solution is organized. It is possible that coordination is not constrained to a specific configuration space, and variables in the task and joint spaces could potentially be coupled. This notion could lead the way to a solution that uses time-delays in task-space to determine delays in joint-space. Note that this is a direct consequence of the specific selected reference frame, as will be discussed in the next section.

In the decomposition model used here time delays were used, but in some other kinematic and electromyography decomposition models, temporal delays are not used. However, the introduction of time delays reduces the number of temporal motion primitives needed to fit the movements of separate d.f. or the movement as a whole. Namely, when fitting kinematic data with a few sources and their corresponding time delays, it is quite likely that many more sources and amplitude coefficients would have been needed to achieve the same fit quality without delays. For instance, a physiological implementation to achieve stable coordination can be to use a small number of central pattern generators (CPGs) combined with delayed coupling, rather than non-delayed CPGs.

In neural control of movements, analysing CPG mechanisms is central to the understanding of the control of locomotion in animal and humans [59–62]. In general, the CPG is a spinal network of neurons capable of generating a rhythmic pattern of alternate activities between flexor and extensor motoneurons in the same leg with reciprocal activation of homologous motoneurons in the contralateral limb. This intrinsic spinal circuitry has been well described in many invertebrate and vertebrate animals, and is highly conserved even in humans, where greater cortical control of spinal modules is required, working in conjunction with sensory feedback [63,64]. However, the unique characteristics of human walking probably reflect a complex neural mechanism responsible for pattern production. In locomotion, the left–right coordination is assumed by a complex network of excitatory and inhibitory commissural interneurons acting on both motor neurons and inhibitory interneurons of the contralateral side [60,65]. One such mechanism for pattern production was described in the literature. As reviewed in Ijspeert & Daley [66], a series of studies on lamprey swimming by Ijspeert and colleagues deciphered the neuronal and network properties of rhythm generation in the local segmental circuits of the spinal cord, and found that the lamprey spinal cord is composed of approximately 100 segments and each segment contains neural oscillators that are part of the locomotor CPG. These CPG circuits were studied to understand how forward swimming is produced, and one model shows that neurons in the local segmental oscillators project up and down the spinal cord, thus creating couplings between oscillators. Furthermore, it was shown that the most likely mechanism to explain the phase lags between oscillators are asymmetries of inter-oscillator couplings.

Simple sinusoids (as modelled in Barliya *et al.* [49]), or more complicated waveforms generated by the combination of several possibly temporally shifted sources, may serve as a temporal referent to produce rhythmic angles patterns. Such a model could correspond to the higher order rhythmic generation structure that determines the rhythmic output of the system as described in the literature [67,68]. Hardware structures, such as reciprocal inhibitory connections of the CPGs, may directly control the motions of the different limb segments by encoding the harmonics of the elevation angles [65,69]. Studies of intersegmental coordination during human gait have indicated that phase shifts between simple sinusoidal oscillators determine the orientation of the intersegmental plane during simple walking [39,49]. Thus, phase shift modulations may also compensate for increased energetic cost during faster walking [38].

However, the neural circuitry that may underly these time shifts has yet to fully be described. Studies in neurological patients may aid in fully understanding the roles of other cortical and subcortical networks contributing to the time delays between adjacent oscillators. For example, the temporal and spatio-temporal organization of kinematic synergies, but not the spatial organization of the muscle patterns [70], was affected in cerebellar ataxia, suggesting that the cerebellum plays a key role in shaping the spatio-temporal characteristics of kinematic synergies. Similarly another study of intersegmental coordination in cerebellar patients reported abnormalities in the phase shifts between different leg segments, pointing to the cerebellum being critical in these phase shifts (Israeli-Korn *et al.* [40]). Overall, the nature of time delays in the human control of movement still merits further study, and this may ultimately help answer many of the underlying questions regarding to how human movements are generated and coordinated.

### 5.3. Reference frames

Previously, numerous studies [47,48,71,72] showed a high preference for representing arm configurations in an extrinsic, or absolute, reference frame. This preference fits well with the results from our analyses, where the absolute joint-space sources are a good fit for the task-space sources.

Pozzo *et al.* [73] have argued that given gravity is a force that is sensed by both the vestibular and proprioceptive systems, guidance of a body segment in space requires the CNS to use an absolute reference frame within which the external positions and displacements of the whole body could be estimated. In fact, there is evidence that the nervous system does have an internal model of gravity that can inform movements [74].

Similarly, in an extensive review in which a variety of different tasks were considered, Soechting & Flanders [75] noted that in all coordinate systems one axis was defined by the gravitational vertical, and another was defined by a sagittal horizontal axis. This may be owing to the neural control strategy whereby human movements in a three-dimensional world are dominated by the force of gravity and by the effect of visual horizon on motion planning. These authors therefore concluded that this common, spatial frame of reference might aid in the exchange of information between brain regions. Indeed, electrophysiological data obtained from neural recordings in the superior colliculus and motor cortex have suggested that neural activities in cortical areas such as the parietal cortex share information and moreover prefer certain coordinate frames when encoding information concerning visual target position and movement direction (Andersen *et al.* [76]).

Hence, neurophysiological studies conducted in monkeys have suggested that arm movements such as reaching are performed in eye-coordinates rather than arm-coordinates, with such an organization being essential to hand-eye coordination [77]. Additionally, there is some evidence that at least in some brain regions, reaching tasks are represented in body-coordinate frames [78]. However, the literature is quite mixed, with some researchers reporting results that seem to imply a neural preference for representing motion in hand or body-coordinates [79–81], and others reporting preference for eye or world-coordinates [82,83], and still others reporting that multiple coordinate systems may be used for different representations or in different brain regions [84,85]. Perhaps, as was proposed by Andersen *et al.* [76] a shared coordinate system is used, in which differentiated gains are used to modulate or transform sensory and motor information between coordinate frames in eye or body spaces. Our results seem to support the preference for world-coordinates at least in unconstrained arm movements, but this issue should be subjected to further investigation.

Importantly, our results support previous work, where Soechting & Ross [48] and Flanders & Soechting [52] showed that arm movements seem to be represented by yaw and elevation angles. Specifically, it was suggested by Flanders & Soechting [52] that target distance and elevation are used to compute arm elevation, and that target azimuth is used to compute arm yaw. Note the similarity between their results and the results obtained here, where one source controls the vertical variables and a second source controls the horizontal variables. At present, we do not know what is the specific role of the third source, but it is postulated that it accounts for the movement variability between different subjects and cycles.

### 5.4. Compositionality

There is a growing body of literature that supports the idea of compositionality [46,86–88]. Compositionality is a concept, by which rather than controlling individual motion strokes or joint rotations, muscles, or other motor components, the nervous system ‘composes’ complex movements from a repertoire of elementary units or building blocks, known as motor primitives which are concatenated and coupled in both space and time to generate more complex movements. Specifically it has been proposed that at the lower levels of the hierarchy, the existence of such modules or primitives reduces the complexity of the control problem, simplifying both motor control and learning [88–92]. It has further been suggested that such primitives exist at different levels of the motor hierarchy ranging from kinematic primitives at the level of end-effector strokes [86,88], joint kinematics [93–95], joint kinetics [96–99] and muscle synergies. Concerning neural planning and control of movement, those different primitives were proposed to be recruited, tuned, sequenced and combined through dedicated neural mechanisms at the spinal, cortical and subcortical levels. Convincing evidence for the existence of motor primitives was provided by rigorous observations of temporal and spatial regularities at different levels of the control hierarchy. Evidence for the existence of muscle synergies was provided based on animal [43,86] and human studies [100,101] during the performance of different motor tasks and through the development of numerical analysis methods and modelling studies allowing decomposition of muscular activities into spatial and temporal motor primitives. Evidence for the existence of kinematic synergies was provided by a considerable number of studies of locomotion, upper limb and other full body movements. Concerning neural mechanisms for the representation and control of motor primitives, studies have been conducted in different animals reporting regularities at the level of the motor cortex [102,103], spinal interneurons [104–106] and motor neurons [100,107]. Thus, while the evidence for the existence of motor primitives provided at the neural level is relatively more scarce, several reviews have been published in recent years reviewing the recent empirical evidence for the neural basis of the different types of primitives at the different motor hierarchical levels. Thus, for example, Cheung & Seki [92] thoroughly reviewed a large number of studies providing evidence for the neural basis for muscle synergies. Likewise, Giszter *et al.* [108] summarized findings of a growing number of studies reporting new findings on rhythm generation and pattern formation networks within the spinal inter-neuronal premotor circuits.

Returning to our findings, in this study we have focused on kinematic synergies closely related to the kinematic synergies described for human and animal locomotion, but in the context of upper limb movements. The sources we have shown here could be considered as the building blocks for synergies and may support future studies of multi-joint movements. Moreover, the dynamic time dependent primitives may correspond to the phasic muscle synergies recently described by Brambilla *et al.* [109]. However, the question still remains to what extent phasic muscle synergies and their compositionality mainly reflect biomechanical/geometric constraints or provide means for implementing schemes for the resolution of kinematic redundancies through patterns of activation and recruitments of spinal inter-neuronal circuits driving different muscles. An extensive study of locomotion movements in a variety of animals ranging from mammals to birds has demonstrated that similar intersegmental coordination constraints apply to humans and animals alike [43].

Moreover, previous work has shown that for humans already at birth or just shortly after, motor primitives are present in the spinal and supraspinal networks [110,111]. Our results suggest that these motion primitives are represented by means of neuronally represented basis functions or sources [110]. However, the tuning of this network is not complete at birth, and the coefficients for producing movements in either the task or joint spaces are *learned* during maturation until network convergence. Similar ideas appeared for the two-thirds power law, where it was observed that the law substrates exist quite early in development, but the movement trajectories do not converge towards exhibiting their mature characteristics until the age of approximately 12 years old [112]. Additionally, the same suggestion was made for the law of intersegmental coordination during locomotion by Bleyenheuft & Detrembleur [113]. In both cases, it was suggested that the network exists at a very early age, but is tuned over time, as described in the work by Dominici *et al.* [111] where this was evaluated in newborn babies. Future work should further explore such tuning.

Finally, this work provides further evidence that the CNS exploits sources (or basis functions) and an absolute reference frame to devise a strategy for solving the IK problem. Ultimately, the shape of the specific sources used by the nervous system is still a subject of research. In particular, the FADA-based sources we computed here might be close to those represented in the CNS, or they may be closer to pure sinusoids, as proposed by Barliya *et al.* [49] in their oscillator model of the intersegmental coordination. Future work will continue to focus on characterizing the sources and the time shifts between them.

## 6. Conclusion

In this work, we have presented theoretical work based on our kinematic analysis of experimentally recorded human arm movements, showing that a single set of sources could be used to represent movement trajectories in both the task and joint-spaces. In particular, we used this observation to mathematically formulate a solution to the IK problem. We showed that a set of three constituent sources could be extracted from movement data and, depending on the reference frame, could be used to represent both joint and task-space movements. Importantly, our analysis showed that using an external, absolute angle representation yielded significantly closer correlation between the joint and task-space sources, and therefore better transferability. Additionally, we investigated the time-delays between sources and different d.f., and found that there are specific couplings that exist and can be identified through our source decomposition approach. We also found essentially one source related to horizontal (azimuth) variables and one source related to vertical (elevation) variables. Finally, we believe that this work has implications for our understanding of motion and motor coordination controlled by the CNS that further research addressing related questions important for our understanding of neural mechanisms of human movement generation and motor-disorders and pathologies.

## Data Availability

The code is available at the following website: http://www.compsens.uni-tuebingen.de/compsens/index.php/downloads/14-toolkit-test. Data available from the Dryad Digital Repository: https://doi.org/10.5061/dryad.ffbg79d1f [114]. Supplementary material is available online [115].

## Footnotes

1 A linear transformation *L* between normed vector spaces *X* and *Y* for which there exists some *M* > 0 such that for all *v* ∈ *X*, ‖*Lv*‖_*Y*_ ≤ *M*‖*v*‖_*X*_.

2 Hereafter, all average values will be accompanied with standard deviation (s.d.) values, unless mentioned otherwise and the sign (s.d.) will be omitted.
